# Identification of heat-treated lithic artifacts via quantitative surface gloss characterization

**DOI:** 10.1038/s41598-026-44878-7

**Published:** 2026-04-02

**Authors:** Robert Stárek, Yonatan Sahle, Balemwal Atnafu, Martin Moník

**Affiliations:** 1 Department of Optics, Palacký University, 17. listopadu 1192/12, 77900 Olomouc, Czech Republic; 2 Department of Archaeology, University of Cape Town, 7701 Rondebosch, South Africa; 3 Department of History and Heritage Management, Arba Minch University, PO Box 21, Arba Minch, Ethiopia; 4 School of Earth Sciences, Addis Ababa University, PO Box 1176, Addis Ababa, Ethiopia; 5 Department of Geology, Palacký University, 17. listopadu 1192/12, 77900 Olomouc, Czech Republic

**Keywords:** Engineering, Materials science

## Abstract

Heat-treated siliceous artifacts from archaeological contexts provide valuable insights into the raw material economy and cognitive prowess of hominins responsible for such a sophisticated and transformative technology. Successful heat treatment induces molecular and structural changes that enhance the mechanical properties of toolstones, ultimately making them more knappable. Among the measurable transformations attained through heat treatment is a reduction in fracture toughness, which reduces the force required for fracture propagation. The internal changes during heat treatment also lead to significantly reduced surface roughness that is visually evident through a characteristically glossy appearance as a result of the improved microscopic smoothness. Reduced surface roughness (i.e., glossiness) is a reliable proxy for improved homogeneity and brittleness, hence fracture predictability. In this study, we present a simple, cost-effective, highly accurate, and field-ready technique for identifying heat-treated siliceous artifacts by measuring gloss as a function of the reduction in surface roughness. Our approach allows archaeologists to overcome the limitation of flat and opaque surfaces required for measurements by conventional gloss meters.

## Introduction

Despite a long history of habitual stone toolmaking in our lineage^1^, the deliberate modification of stone raw materials through heat treatment (HT) started only 400-200 ka^2^. Toolstone HT represents one of the earliest material-engineering techniques. Intentional thermal alteration of siliceous stones is a complex process that typically requires a degree of planning depth, attention, and knowledge of irreversibly transformative properties. It is thus considered part of the suite of behaviors commonly referred to as complex or “modern”^3–5^. Controversies notwithstanding, such behavioral traits are widely used in discussions surrounding the cognitive capacities of Pleistocene anatomically modern humans relative to their extinct cousins.

The desired outcome of stone HT is material transformation for easier flakeability and improved flaking control^3,6^. There were likely several different techniques of HT used in the past but identifying a specific technique employed at a given archaeological site remains a challenge. Archaeologists have instead long focused on the more fundamental exercise of inferring intentional and successful HT on siliceous archaeological artifacts. Based mainly on experimentaltests^7–15^, and early ethnographic reports^16–18^, several of these studies have employed various methods of recognizing HT lithic artifacts from archaeological contexts, with an increasing emphasis on using non-destructive methods that would conserve the artifacts and allow future analyses. The various approaches along these lines range from the observation in HT lithic artifacts of color changes^19^, (palaeo)magnetic^20^, elemental^21^, structural^22^, or crystallographic^23^ measurements, to mechanical testing^5,24^.

The realization that gloss, or luster, is a good indicator of HT was made early on, although its relationship with improved fracture propagation and predictability was more explicitly established later^25–32^. The presence of gloss implies smoother surfaces and sharper edges of the desired knapping products^33,34^. Despite this, gloss intensity has rarely been quantified using instrumental methods in studies of HT. We present here a novel approach for the identification of HT by quantifying gloss on the flaked surfaces of ethnographically HT chalcedony toolstones^34^. Our technique identifies, with high levels of accuracy, HT samples even on translucent/partially transparent samples with great variation in surface roughness depending on their HT status. Its simplicity, reproducibility, reliability, and cost-effectiveness make this method suitable for a wide range of users and settings, including in field laboratories.

### Assessing surface morphology and gloss

In the optical assessment of surface morphology, confocal microscopy, or one of its profilometric modalities, has proved to be reliable for determining surface roughness^35,36^. By scanning a focused beam over the surface, a three-dimensional surface profile can be reconstructed in this method, allowing the assessment of both waviness and roughness. Capturing typical height variations (from a few hundred nanometers to several micrometers) requires high transverse and longitudinal resolution, which is achieved using high-numerical-aperture (NA) objectives. However, these objectives typically operate at short working distances, making surface observation less practical. Additionally, only a small portion of the surface can be analyzed with this technique, as the scanning range decreases inversely with increasing magnification. Finally, confocal microscopes require high-quality optics, precise and fast scanning mechanisms, and sensitive high-speed detectors, making them expensive and not easily portable.

Surface roughness can also be measured using laser speckle metrology^37^. In this family of methods, a beam of highly coherent light is diffused by the rough surface, creating a speckle pattern detectable by a digital camera. This pattern encodes information about the surface roughness. While effective for flat, opaque surfaces, the measurement of very rough surfaces, such as those of rocks, is challenging. Specifically, once the surface roughness exceeds a certain fraction of the wavelength, the speckle contrast saturates and becomes insensitive to higher values of roughness. Another complication in the measurement of wavy surfaces is that each surface element is effectively viewed from a different angle, complicating direct comparison. A final complication arises in the analysis of non-opaque (i.e., translucent or semi-transparent) materials that produce multiple scattering paths, which makes the camera capture a complex and unpredictable mixture of speckle patterns.

Interferometry represents an alternative high-precision method for measuring surface profiles^ 38^, and can achieve sub-wavelength measurement precision. Here, an optical probe wave reflected from the surface encodes height information in its phase. When this probe is optically mixed with a reference wave, the intensity of the resulting interference pattern reveals the phase difference. However, the direct determination of optical path difference is ambiguous because the interferometric intensity is a periodic function of a phase difference. To resolve this ambiguity, phase unwrapping is used, assuming a smoothly varying surface profile, which is not a valid assumption for most lithic artifacts^34^. Alternative methods, such as multi-wavelength interferometry^39^, must be used to collect unambiguous measurements.

An edge case of this multi-wavelength approach is profilometry based on optical coherence tomography (OCT)^40^. In OCT, light with a broad spectrum is used for both the reference and signal beams. These beams interfere only when their relative time delay is within the coherence time, which shortens with broader spectra. While commercial OCT systems achieve micrometer-scale precision, this is insufficient for sub-micron roughness measurements. Moreover, OCT systems require either highly precise mechanical mirror scanning, beam scanning across the surface, or swept-source technology, each reducing cost-efficiency. A further challenge is the high diffusivity of unheated rock samples, which scatters most light outside the aperture of long-working-distance optics. Consequently, the signal wave is often comparable in magnitude to residual reflections from other optical components, degrading measurement quality.

The microscopic profile of a surface directly influences the way it reflects the light at a macroscopic level^41–44^. Smooth surfaces with sub-wavelength local variations act effectively as a mirror, and rough surfaces act as a diffuser. Assessment of the gloss (luster) of knapped lithic surfaces thus readily provides information on the surface roughness. Although the gloss of the surface can be observed with the naked eye^34^, objective and quantitative assessments, i.e., measurements, can provide invaluable information. A typical gloss meter illuminates a small surface area from a defined angle and measures the intensity of the specular reflection^45^. The reflected intensity gauges the gloss; a glossy surface reflects most of the beam’s energy specularly to the detector, while matte surfaces scatter it to a broad distribution of angles. The detector reading is typically compared to values measured on reference samples. However, such gloss measurement will be challenging to collect on the knapped surfaces of HT siliceous rocks, such as chalcedony, because gloss meters typically require flat surfaces for reliable and repeatable gloss determination. Even with gloss meters adapted for the measurement of curved surfaces, it is not evident how their readings can be interpreted, as these are related to standard reference samples that are unrelated to the materials being measured. It thus remains difficult to apply the measured values from such techniques for the identification of HT on materials such as chalcedony.

We addressed these problems by developing a simple optical method adapted for the quantitative assessment of gloss from the knapped surfaces of chalcedony. Our approach is tolerant to surface waviness, requires no scanning mechanism, and operates using consumer-grade cameras and a standard laser pointer. Using ethnographic samples with known HT processes and material transformations^34^, we demonstrate that our novel approach reliably distinguishes between heat-treated (HT) and unheated (unHT) samples. In addition to its classification accuracy and cost-effectiveness, the technique can also be applied to other fine-grained SiO$\phantom{0}_2$-rich lithologies.

## Results

**Fig. 1:**
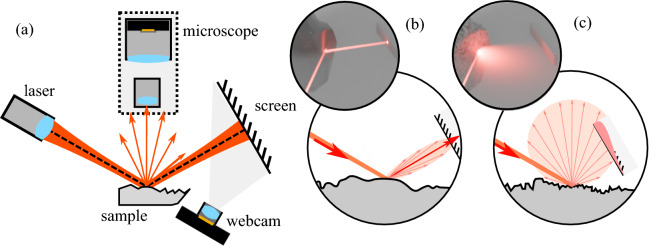
Schematics of the measurement setup (**a**) depicted from the top, and two edge cases of specular (**b**) and diffuse (**c**) reflection. In panels (**b**) and (**c**), the large red arrow indicates the incident laser beam, and the smaller arrows indicate the reflection and represent the intensity by their length. Red distribution curves, plotted on the screen, show the light intensity there. A 3D rendering in the top section of panels (**b**) and (**c**) illustrates the situation further.

To determine gloss using our approach, the rock sample is illuminated by a focused laser beam, as illustrated in Figure 1(a). The laser spot has a diameter of approximately 0.2 mm on the sample surface. At this scale, the surface waviness can be neglected, and the surface is approximated as locally flat. The angle of incidence is 62 degrees. The laser beam is reflected onto a square paper screen. The screen is positioned such that the specular reflection appears at its center. A web camera captures an image of the screen which is then processed digitally. See Methods for more information on the geometry of the optical setup, calibration and data processing procedures. This image contains information about the reflectivity of the sample, as the smooth surfaces reflect light efficiently and do not distort the laser beam, while rough surfaces distort the reflected beam heavily and spread the light into a broad angular distribution, as visualized in panels (b) and (c).

**Fig. 2:**
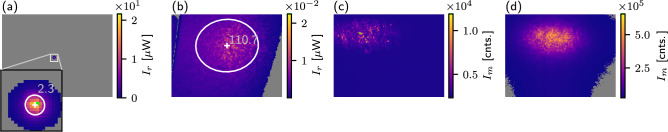
Panels (**a**) and (**b**) show screen photographs capturing a specular reflection from a thin cover glass (**a**) and a diffuse reflection from the office paper (**b**). Panels (**c**) and (**d**) show the laser light scattered from the glass (**c**) and paper (**d**) surface and collected by the optical microscope. False colors indicate the camera’s exposure-corrected response; underexposed areas are plotted in gray, and overexposed regions are plotted in green, and for each remaining color, the numerical value can be assigned using the corresponding color bars. The false colors in panels (**a,b**) represent optical power per pixel, $I_r$, in screen photograph, while in panels (**c,d**), they show the exposure-corrected pixel intensity in the microphotograph, $I_m$, in units of camera counts. The multipliers above the color bars correspond to the tick values, e.g., the yellow color in panel (**a**) encodes value 20 $\mu$W. Images in panels (**a**) and (**b**) show a 55$\times$45-mm-area of the screen and the images in panels (c,d) have field of view of 0.7$\times$0.5 mm. The white ellipses in (**a**) and (**b**) indicate the spread of the reflected spot, and the numbers represent the mean semi-axis. The inset in panel (**a**) zooms in on a $2 \times 2$ mm area ($20 \times 20$ pixels) centered around the reflected laser spot and the color scale is shared with the main heatmap in panel (**a**).

To capture two extreme cases, we replaced the lithic sample with a microscope cover glass, producing a bright, well-localized reflection spot, and then with a piece of standard copy paper, resulting in a low-intensity speckle pattern that covers the entire screen. These edge cases of reflection from a flat thin cover glass and paper are shown in Figure 2(a,b). Due to its small thickness of 0.17 mm, the double reflection from the cover glass is not apparent in the camera image, such that both reflected spots mostly overlap.

In contrast to using a single small detector aligned with the expected direction of specular reflection, our nuanced approach significantly reduces sensitivity to the exact sample placement, particularly the angle of incidence. The resultant screen image reveals the direction of reflection and shows the extent of light diffusion due to surface roughness. Since the intensity of light scattered from the screen to the camera can vary by several orders of magnitude, we enhance the dynamic range by acquiring the camera images at multiple exposure times (see Methods for details on calibration).

An auxiliary optical microscope, comprising a 15 mm lens, a 150 mm tube lens, and a second camera, was used to ensure proper sample placement and to select representative, locally flat surface areas, thereby avoiding uncharacteristic defects. The limited depth of field, approximately 0.05 mm, helps to place the sample consistently, as tilting would result in defocused areas in the image. When the sample is illuminated by the laser, some scattered light is captured by this auxiliary microscope, providing additional information on the diffusivity of the sample. In our experiments, glass appeared dark under laser illumination, whereas paper appeared bright, as shown in Figure 2(c, d).

**Fig. 3:**
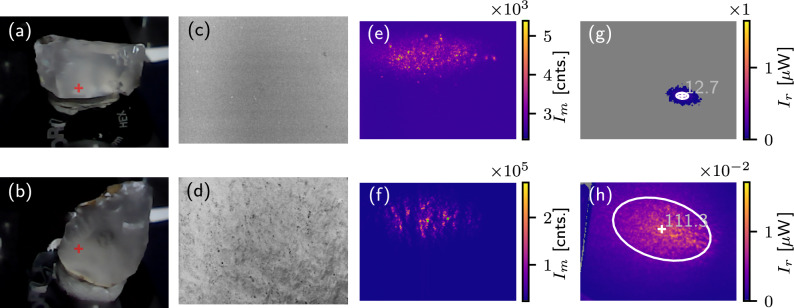
A comparison of the HT (**a,c,e,g**) to the unHT (**b,d,f,h**) sample. The macro photographs are shown in panels (**a**) and (**b**). The red mark indicates where the laser impinges on the surface, and microphotographs of these areas are shown in panels (**c**) and (**d**) under LED illumination. Panels (**e**) and (**f**) show microphotographs of the same area illuminated by a laser spot. Finally, panels (**g**) and (**h**) show screen photographs with the reflected laser. The color coding in panels (**e,f,g,h**) is organized in the same way as in Fig. 2.

In addition to glass and paper, we captured an overview image of chalcedony samples using another webcam, as well as a microphotograph of the surface under broad LED illumination. The raw data from typical HT and unHT samples are shown in Figure 3. From the collected images, several metrics were computed to characterize the gloss of each sample. First, we estimated the intensity, both maximum and total, of light scattered on the screen as well as the size of the reflected laser spot. From these values, we calculated the energy density of the reflection. We also computed the entropy of the screen image to quantify the spatial distribution of reflected light. Furthermore, we calculated the total intensity of laser light collected by the microscope. Mathematical definitions of these metrics are provided in the Methods section.

We tested the method on six HT and six unHT (i.e., reference) chalcedony samples from Konso, Ethiopia, where contemporary hideworkers use traditional techniques of thermally improving the flaking qualities of their toolstones. The successful material transformations of the Konso chalcedony HT were previously documented using various methods, including rugosimetric analysis^34^. The macrophotographs of all samples studied here are provided in the open dataset^46^.

**Fig. 4:**
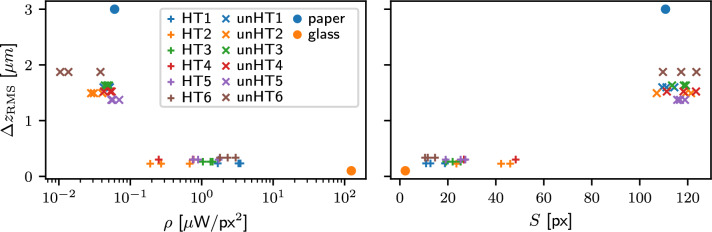
Correlation between RMS surface roughness $\Delta z_{\textrm{RMS}}$ and energy density $\rho$ seen on screen (**a**), and spread *S* of the laser spot on screen (**b**). Heat-treated (HT) and unheated (unHT) categories are marked by crosses (’+’) and $\times$-symbols, respectively. Color is used to discriminate between samples within each category, while individual measurements of a single sample are distinguished by shading. Solid dots represent the reference cases. Surface roughness of the paper is adopted from^47^.

Each sample was measured at three randomly selected typical loci on the same surface. The most significant image-derived metrics from our approach were compared with RMS surface roughness values obtained using a scientific-grade optical confocal microscope, see Methods for more information about this reference measurement. A certain correlation is observed between surface roughness and the metrics characterizing laser reflection, demonstrating that both types of measurements can distinguish between HT and unHT samples (Fig. 4).

For each sample, a single surface roughness value was calculated as the mean of three measurements taken at different loci. Since the surface roughness and gloss data were acquired from different regions of the same surface, we cannot interpret the observed correlation as a calibration curve. Despite this limitation, the results illustrate the general trend. In future experiments, referencing techniques such as gluing reference beads^ 48^ could be used to consistently measure the same region with multiple devices. The gloss-related data for the three trials for a single sample are reported separately to illustrate the variability of the measurement.

The measurements collected from the 36 loci on the 12 samples served as a reference dataset. To determine which metric obtained is the most useful for determining HT, we used linear discriminant analysis^49^. Based on the discriminant coefficients obtained, we were able to completely separate the two classes of samples. The most important factors in the discrimination were the energy maximum, the spread of the laser spot, and the corresponding density in the obtained specular reflection data. The other important measure was the image entropy of the diffuse light seen by the microscope. Interestingly, the maximum value of the diffuse reflection was not a key factor. Even a very small locally flat surface element can reflect incident laser light directly into the microscope, thus producing a very large value in an otherwise dim image. We believe that this is the reason why the image diffuse reflection data have less weight in the discrimination process. In order to study the ability to discriminate between unHT and HT samples using the gloss readings alone, we avoided using the profilometry data for discrimination purposes. This mimics a situation in which the profilometer is unavailable to the analyst. Previous studies^34,50^ have shown that the profilometry provides valuable data for this discrimination.

To test classification consistency, we randomly selected two samples from each group for repeated measurements. The classification succeeded in all cases. We also evaluated the discrimination on one previously unexamined HT sample and one previously unexamined unHT sample, each of which was measured at three random loci. For these new measurements, the classifier consistently produced correct predictions. Although the classification was successful in 18 tested cases, the reliability of the classification is not necessarily perfect. We estimate the lower bound by formulating a Bayesian inference problem to determine the success probability conditioned on our observations. With the choice of the Jeffreys prior^51^, we estimate that the probability of correct classification is at least 90% with a confidence level of 95%.

To summarize, our data confirm that the knapped surfaces of HT samples are significantly glossier than those of unHT samples..

## Discussion

Archaeologists increasingly rely on sundry analytical techniques from various fields of study in order to make strong inferences about past human technological and other behaviors. At the center of recent methodological advances sits the need for objective, if not quantifiable, data collected using efficient and non-invasive methods. As part of such a growing endeavor, our novel approach objectively characterizes the optical gloss of chalcedony flakes to determine whether they have undergone successful HT before knapping. The measurable data from our analysis indicate that the optical gloss of a knapped surface is a reliable indicator of HT, as the specularly reflected energy density typically differs by two orders of magnitude. This is further demonstrated by a direct correspondence between optical gloss and surface roughness parameters, relating our findings to results from optical surface profilometry.

When identifying HT artifacts in archaeological contexts, the potential influence of taphonomic processes must always be taken into account. *Surface* modifications resulting from patination, aeolian activity, soil or sand abrasion, and use-wear can generate surface gloss comparable to that produced by HT^9,32,52^. Such processes do not improve raw material flaking properties, as they do not modify the material structure within its volume, but are likely to affect laser-beam interaction in a manner similar to that observed on thermally altered surfaces. Consequently, the identification of HT cannot rely on a single diagnostic criterion; rather, it must be supported by multiple lines of evidence. These include, in particular, contrasts between pre- and post-HT removal surfaces, the presence of tempering residues (e.g., carbonized plant remains associated with the HT process) on HT blanks, and evidence from the lithic operational sequence indicating knowledge of pressure flaking technology or the intentional application of HT^32,52,53^. The intentionality and careful control behind the Konso HT process^ 34^, for example, would be indicated by heating pits and associated insulation material, chalcedony nodules with gloss contrast, large amounts of lustrous flakes, and significantly improved mechanical properties of the HT chalcedony.

The identification of HT in siliceous artifacts from archaeological sites commonly involves measurements of their mechanical, structural, crystallographic, and/or elemental attributes. Most of these analyses require specialized instrumentation, which limits their applicability to materials housed across several museums. In the extreme cases, not only must the artifacts of interest be loaned from their repositories, but they must sometimes also be (partially-)destroyed. Cost-effective, widely applicable, and non-invasive approaches using portable but accurate measurement instruments are thus imperative for such studies. The simple optical setup we have introduced here is capable of quantitatively capturing gloss for samples with significant variations in surface roughness and, unlike standard gloss meters, for wavy surfaces as well. Although the current repeatability of the angle of incidence and the standard linear discrimination analysis of our approach is sufficient for the identification of HT on siliceous rocks, the angular distribution of the reflected light can also be further determined with careful calibration and better control over the angle of incidence. Despite a strong correlation, and admittedly surprisingly, the microscope images of the surface illuminated by laser light (i.e., the diffuse-reflectivity data) alone were not a reliable indicator of HT. We hypothesize that even a tiny, locally flat area oriented to reflect light into the microscope could create a high intensity in the image and increase the uncertainty of such a determination.

Given that gloss is a very common and easily observable feature of HT artifacts, we expect that our cost-effective, field-ready, and reliable approach will not only advance research in the area but also provide a crucial alternative for materials that must be analyzed non-invasively and while housed in their repositories. As a versatile approach, the measurement apparatus can be further simplified by removing the optical microscope and the auxiliary webcam. It can also be cost-optimized by using a focusable laser pointer instead of a fiber-coupled laser. Moreover, since the apparatus relies on imaging, it is tolerant of small mechanical instabilities. These features make the proposed approach to gloss measurement a strong candidate for field use or entry-level equipment. Although HT Konso chalcedony flakes can often be distinguished from unHT specimens by the naked eye, reliable identification and quantification of surface gloss or gloss contrast, as well as objective discrimination between HT and unHT pieces, are only achievable through analytical methods such as the one developed in this study. The fact that our apparatus is relatively easy to assemble means that it is ideal for analogical measurements on other siliceous archaeological artifacts. Although our analysis has only been conducted on hydrothermal chalcedony, the appearance of luster on HT and flaked artifacts made of chert, flint, or silcrete^5,32,53–55^ indicates that the method can be applied to any SiO$\phantom{0}_2$-rich fine-grained raw material.

We observed that the unHT samples are characterized by their rough texture seen in the microphotographs. Therefore, as an alternative to direct characterization of the gloss, one could further consider texture analysis. It has also been shown that texture analysis, based on gray-level co-occurrence matrix^56^ or machine-learning^57^, of a surface microphotograph can estimate its surface roughness parameters. Recent successes in texture-based classification of use-wear analysis^58–60^, as well as growing open datasets^61^ indicate that machine learning methods are promising for identifying HT of lithic artifacts. The presented optical setup may further support such approaches by providing both surface texture images and reflection measurements that reliably distinguish HT and unHT samples, as demonstrated here.

## Methods

### Sample preparation

The studied chalcedony samples were obtained from the Ubaba quarry located in the Konso Zone, Ethiopia. Before heat treatment (HT), a part of the sample was separated as an unheated (unHT) reference sample.

HT was performed traditionally in dedicated pits indoors. The pit was preheated by the ambient rise in temperature from the hearth fire, about 20 cm away, before the chalcedony nodules were placed inside. The pit was then insulated with layers of wool and sealed with a piece of broken pottery. Hot coals, ash, and embers were placed on top of the pottery cover to provide consistent heat to the toolstones in the pit for 24–48 hour s^34^.

The consistent pit temperature measures *ca*. $350-390^{\circ }$C across several hours before the toolstones are removed from the pit and allowed to cool near the hearth but away from the ambient heat. This allows for slow cooling, usually overnight, and avoids crazing or fracturing from thermal shock. The result is successfully HT chalcedony toolstones with a visibly lustrous and waxy look, a smoother surface, significantly reduced fracture toughness, and increased brittleness, all of which result in the desired ease and success in removing smooth and untruncated blades for modification into scrapers^34^.

### Experimental setup

**Fig. 5:**
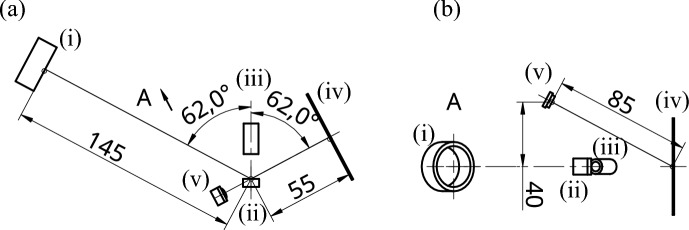
Top (**a**) and projected (**b**) views of the apparatus geometry. The laser propagates from the 150-mm focusing lens (i) in the direction of the arrow to the sample (ii), where it is scattered to the 15-mm lens objective (iii) of the auxiliary microscope. The reflected laser beam hits the screen (iv), which is photographed by the NoIR camera (v), situated above the sample. Dimensions are given in millimeters, and angles are given in degrees.

To illuminate the sample with the laser beam, we used a fiber-coupled infrared 810 nm laser diode, which we decoupled into free space using an 11 mm lens and subsequently attenuated to a power level of a few hundred $\mu$W using a neutral-density filter. The laser beam was focused on the sample using a 150-mm plano-convex lens Thorlabs LA-1433-B. To obtain surface microphotographs, we used an LED illumination with a central wavelength of 810 nm. The optical microscope consisted of a 15-mm molded aspherical lens (Thorlabs C260TM-A) as an objective, a 150-mm tube lens (Thorlabs LA-1433-B), and a standard industrial monochromatic CMOS camera (DMK 23U74). The sample was mounted on a linear stage equipped with a fine-threaded screw for precise focusing. The screen, consisting of standard office paper attached to a holder, was photographed using a low-cost camera module (Raspberry Pi Camera NoIR V2) from a distance of 85 mm, placed 40 mm above the plane of laser incidence. The geometrical relations are sketched in Figure 5. The microscope’s limited depth of field aids in the precision of sample placement. Misplacing the sample longitudinally by 0.05 mm results in a blurred image. The limited depth of field also ensures consistent angular orientation. Tilting the sample by 3 degrees from the optical axis is noticeable as well.

On the other hand, the setup is largely unaffected by the limited placement precision of the remaining components. The tolerance is based on the geometry being set once during construction and then staying constant throughout calibration and data acquisition, ensuring comparable data. The focusing lens can tolerate a few millimeters of displacement because the depth of focus is measured in centimeters. A few-degree systematic angular error is not critical since all samples are measured and compared using the same angle. The sample-screen distance affects the detectable solid angle for diffused light. As shown in Fig. 5, a smaller distance means a larger detectable solid angle. Therefore, we placed the screen as close to the sample as possible. A few-mm systematic error in this distance is not a significant obstacle. However, tilting the screen is undesirable because it would distort and elongate the reflected laser spot perceived by the camera. We aligned the screen using a strong laser reflection from a mirror to minimize the lateral size of the laser spot. At a minimum, the screen is perpendicular to the laser beam. The distance between the NoIR camera and the screen determines the perspective and the camera’s response to the optical power incident on the screen. These are addressed by the calibration described in the next section.

Above the microscope objective, we attached another low-cost web camera to aid in the coarse positioning of the sample. The image acquisition sequence is controlled from a computer and comprises taking a microphotograph using LED illumination, another microphotograph under laser illumination, and a photograph of the screen. All acquisitions were performed with multiple exposure times to accommodate a wide range of possible detected intensities. The reference photo of the sample, taken with a web camera, concludes the sequence. The acquisition was done in a dark room with the main room lights switched off. In cases where the darkness cannot be achieved in the room, a black box might be used instead to block ambient light. Alternatively, spectrally narrow band-pass filters can be placed in front of light sensors to block the ambient light, while keeping the infrared signal.

### Device calibration and data processing

**Fig. 6:**
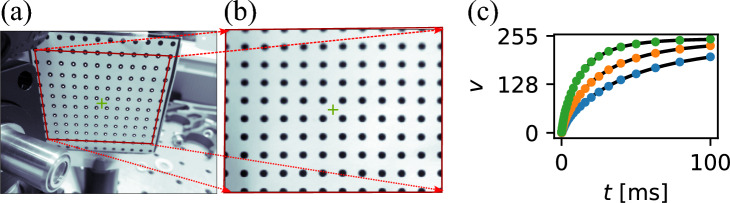
Perspective (**a**, **b**) and power (**c**) calibration of the screen camera. A photograph of a calibration grid (**a**) and the corresponding result of the perspective-compensating transformation (**b**). The camera’s nonlinear response (**c**) shows that the value of the brightest pixel, *v*, as a function of exposure time, *t*, follows an exponential saturation curve. The response was calibrated at power levels of 1, 2, and 4 $\mu$W, shown as blue, orange, and green points, respectively. Black lines represent the fitted empirical model.

To analyze the optical power distribution reflected, we use a photograph of the screen. To compensate for perspective distortion caused by the placement of the web camera, we compute a suitable affine transformation. To determine this transformation, we attach a grid of dots with 5-mm spacing to the screen and take a photograph, as shown in Figure 6(a). We identify the region of interest (ROI) and, within it, localize the dots. Given their known coordinates in both image space and real-world space, we use the *opencv2* library to compute and apply the transformation. The result of the transformation, implemented using the *undistort* and *warpPerspective* functions, is shown in panel (b). The pixel coordinates in the transformed image are directly related to the real-world coordinates.

Now, we relate the camera response to the actual optical power, so the optical power incident on the screen can be estimated from the camera signal. The relationship between the camera signal and the actual optical power on the screen depends on the distance between the screen and the web camera, as well as the web camera’s sensitivity and the gain and exposure settings used. We take these parameters into account using the following procedure. Thanks to the calibration procedure, we can tolerate limited placement precision of the screen and web camera. We have performed power calibration by introducing a normally incident laser beam of several total powers to the screen and, for each, taking the screen photographs, varying the exposure time. An example of calibration data is shown in Fig. 6. The camera response value as a function of the exposure time is fitted with the following function

1 $$\begin{aligned} v = a\left( 1 - e^{-\left( \frac{t}{b}\right) ^{0.8}}\right) +c. \end{aligned}$$

From the available fits, we have composed the following empirical formula describing the relation between optical power *I* in the area corresponding to a pixel on the screen and the corresponding pixel value *v*

2 $$\begin{aligned} I = g \cdot B \cdot \left| \ln \left( 1 - \frac{v}{255} \right) \right| ^{\frac{10}{8}}, \end{aligned}$$

where $g=0.04$ is the geometric factor that relates the size of the pixels in real space with the spatial profile of the calibration laser, and $B \approx 3.58 \times 10^4 \cdot t^{-0.954}$, and *t* is the exposure time in microseconds. These coefficients were determined using the least-squares fitting of the calibration data, and their numerical values are specific to the actual geometry of the experimental setup, as well as the camera sensitivity. The need for power calibration could be effectively circumvented by using a scientific camera with a linearized response. Note that the same power incident on the left and right edges of the screen results in various responses at the camera. This is due to the varying distance of the spot on screen to the camera and the varying angle of incidence as well. Given the large differences in reflected optical power between HT and unHT samples, we can neglect this effect. The spatial dependence could be, in principle, accounted for with additional calibration.

Given the knowledge of the sample and screen positions in the laboratory reference frame, one can, in principle, use these calibrations to interpret the screen images capturing diffused and reflected light as a spatial distribution. With the knowledge of the angle of incidence, one can convert this spatial distribution into an angular distribution of reflected optical power. Fortunately, neither absolute power nor angular calibration is necessary to distinguish between an HT and an unHT sample, provided the power density readings are consistent across the samples.

To estimate the size of the reflected spot in the screen photograph, we first set a reference pixel value $v_r$ such that pixels with values greater than $v_r$ contribute to 75% of the total power in the screen photograph. We then subtract the value $v_r$ from the image, clip it to positive values, and apply the L1 norm. This procedure effectively performs background subtraction. The resulting image is then interpreted as a 2D probability distribution. The highest and lowest spreads are determined as the square roots of the corresponding covariance matrix eigenvalues, while the eigenvectors determine the directions. The centroid of the reflected spot is also determined as a reference. These metrics can be visualized as an ellipse surrounding the reflected spot. The total optical power is calculated as the sum of the pixel intensity values within this ellipse. The energy density measure is then calculated as $\rho = E_t/{S}^2$, where *S* is the mean spread of the reflected spot (in pixels) and $E_t$ is the total optical power within the spot.

### Reference measurements

The reference profilometric data were obtained as part of Ref^ 34^. using a Sensofar S-Neox profilometer based on an optical confocal microscope. The profilometer used white light and a long-working distance objective of 50$\times$ magnification, with a numerical aperture of 0.45 and a spatial sampling of 0.26$\mu$m.

We chose the RMS height, also known as the $S_q$ parameter, as a representative measure of the surface roughness. The other measures of surface roughness linearly correlate with the RMS height with a high coefficient of determination $R^2$. We investigated the following parameters: maximum peak and pit height ($S_p$, $S_v$), arithmetic mean height ($S_a$), maximum height ($S_z$), core roughness depth ($S_k$), reduced peak and dale height ($S_{pk}$, $S_{vk}$), maximum depth of furrows (MaDF), and mean depth of furrows (MeDF). The lowest and the highest coefficient of determinations are $R^{2} = 0.88$ for the $S_p$ parameter, and $R^2 = 0.998$ for the $S_a$ parameter, respectively. The full rugosimetry data are available in the Supplemental Material of our previous study^34^. Each sample was measured three times at different locations, and the mean reading is used. We then measured the samples with the tested method near these places.

## Data Availability

All data acquired during this study and the corresponding scripts for their processing are provided in the public repository https://zenodo.org/records/17546043
